# 

**Published:** 1812-03

**Authors:** 


### MONTHLY CATALOGUE OF MEDICAL BOOKS.

AN Essay on Scrophula, in which an Account of the Effect of the Carbonas Ammoniæ, as a Remedy in that disease, is submitted to the medical profession. By Charles Armstrong, M. D. 8vo.—Cadell and Davies.

Letters to a Student of Medicine, on his commencing Practice, containing a comparison of the condition of naval, military, and private, Practitioners, with a few Directions for Medical Gentlemen who propose entering the Royal Navy. By John Strang. 8vo.—Mawman.

Narrative respecting the Case of a confirmed Cancer, which was speedily and radically cured by an external Application only, without the chirurgical operation of Cutting, or the use of Medicines internally. 8vo. sewed.—Murray.

Cases of Apoplexy and Lethargy, with Observations upon the Comatose Disease. By J. Cheyne, M.D. 8vo. boards.—Underwood.

A Report on the Medicinal Effects of an Alluminous Chalybeate Water, lately discovered at Sand-rocks, in the parish of Chale, Isle of Wight, pointing out its Efficacy in the Walcheren and other Diseases incident to Soldiers who have served abroad; and more particularly the advantages to be derived from its Introduction into private Practice. 8vo. boards.—Mawman.

Observations on the Contracted Intestinum Rectum, and the Mode of Treatment, &c. By W. White, Surgeon, Bath. 12mo.—Bath printed.

### *NOTICES TO CORRESPONDENTS.*

*W. H. Leadbeater, Overton, Hants, requests to be informed, through the medium of the Journal, or per Post, whether a Medical Practitioner within Sixty Miles of London can be admitted a Member of the Society for the relief of the Widows and Orphans of Medical Men in London and its vicinity?*

*We gratefully acknowledge favors from D. Thompson, M.D.; George Nesse Hill; Mr. Hammick; L. N.; M. R. C. S.; &c. &c.*

*The Review of Mr. Muter's Work, unavoidably, from a pressure of matter, postponed, will appear in our next.*

